# Delayed uterine rupture and septic shock after high-intensity focused ultrasound for uterine fibroids: a case report

**DOI:** 10.3389/fonc.2025.1697568

**Published:** 2025-12-08

**Authors:** Huan Xia, Wenyin Liu, Hui Mao, Chi Li, Yizheng Zu, Yi Yu, Wanying Chen, Yaoyang Zhang, Jiming Chen

**Affiliations:** 1 Department of Obstetrics and Gynecology, Zigong Fourth People’s Hospital, Zigong, Sichuan, China; 2 Department of Gynecology, The People’s Hospital of Wenshan Zhuang and Miao Autonomous Prefecture, Wenshan, Yunnan, China; 3 Department of Gynecology, Changzhou Second People’s Hospital, The Third Affiliated Hospital of Nanjing Medical University, Changzhou, Jiangsu, China

**Keywords:** high-intensity focused ultrasound, uterine fibroids, uterine rupture, septic shock, HIFU

## Abstract

**Background:**

High-intensity focused ultrasound (HIFU) ablation is widely regarded as a safe and minimally invasive technique for treating uterine fibroids. However, severe complications such as uterine rupture and septic shock remain exceedingly rare.

**Case presentation:**

We report a case of a 42-year-old woman who developed uterine rupture complicated by septic shock one month after undergoing HIFU ablation for uterine fibroids.

**Conclusions:**

Although HIFU ablation for uterine fibroids is widely considered a safe and minimally invasive procedure, clinicians should recognize the potential for severe complications, including uterine rupture and septic shock.

## 1 Introduction

Uterine fibroids, as one of the most common benign tumors in women of reproductive age, have a prevalence rate as high as 20%–40% (1). They often lead to abnormal menstruation, anemia, and pelvic compression symptoms, significantly impacting patients’ quality of life. In recent years, HIFU has emerged as a non-invasive treatment technology. With advantages such as precise ablation of lesions, preservation of uterine function, and rapid postoperative recovery, it has gradually become an important treatment option for symptomatic uterine fibroids (2–5). Multiple clinical studies have confirmed that HIFU is highly effective in alleviating symptoms and reducing fibroid volume, with a favorable overall safety profile and a lower incidence of severe complications compared to traditional surgery (6–8).

However, as the application of HIFU becomes more widespread, its potential risks require careful evaluation (9). In the existing literature, reported complications of HIFU are mostly limited to skin burns, nerve injuries, or short-term pain (10, 11). Severe complications such as uterine perforation and septic shock are extremely rare, and their underlying mechanisms and risk factors remain poorly understood. In this case, a 42-year-old woman developed sudden-onset peritonitis, uterine rupture, and septic shock one month after undergoing HIFU treatment for uterine fibroids, ultimately requiring a total hysterectomy. The occurrence of this rare complication suggests that, although HIFU technology is minimally invasive, the long-term effects of thermal damage on uterine tissue, the risk of postoperative infection, and the need for individualized patient assessment still require in-depth investigation.

## 2 Case presentation

### 2.1 Chief medical history

The patient is a 42-year-old female with a BMI of 26.6 kg/m². On December 23, 2024, she presented to our hospital with increased menstrual flow and prolonged menstrual periods. Following investigations including blood analysis and pelvic MRI, she was diagnosed with: Uterine Leiomyoma and Severe Anemia. Starting December 23, 2024, she received 4.5 units of leukocyte-depleted packed red blood cells (Type O, Rh-positive) intravenously to correct the anemia. On December 27, 2024, she HIFU ablation for the uterine fibroid. On-board ultrasound findings: The uterus was anteverted. A hypoechoic mass measuring approximately 90×93×83 mm, suggestive of a fibroid, was seen in the anterior uterine wall. Post-treatment, the lesion showed conglomerate grayscale changes. HIFU procedure details: After satisfactory bladder filling adjustment and pre-operative targeting, the free-hand treatment mode was selected. The focal point was initially placed slightly towards the foot side of the center of the anterior wall fibroid. Irradiation was performed at 400W, 1:3 duty cycle, for 3 passes. The patient tolerated the procedure poorly, frequently reporting skin burning sensation and lower abdominal distending pain. Intermittent bladder emptying was performed, and irradiation was delivered slowly. After patient tolerance improved somewhat, the treatment dose was increased to 400W, 2:4 duty cycle, for 2 passes. Layered, small-area spot irradiation was performed. At 356 seconds, conglomerate echogenic changes appeared within the lesion. Layered “leading” of the grayscale change was performed, with satisfactory spread of the grayscale mass. Skin cooling breaks were taken every 60 seconds. Irradiation was continued until 1,027 seconds, by which the grayscale change within the lesion had fully developed and spread. Contrast-enhanced imaging confirmed satisfactory necrosis of the treated lesion.

### 2.2 Post-HIFU course

The patient experienced moderate lower abdominal pain and discomfort after the HIFU procedure, which was treated with oral Ibuprofen for one week, after which the abdominal pain resolved. Following HIFU treatment, the patient received two courses of subcutaneous Leuprolide Acetate 3.75 mg injections, on December 29, 2024, and January 26, 2025, respectively.

On February 16, 2025, the patient experienced sudden onset of generalized abdominal pain without an obvious precipitating factor. The pain was primarily characterized as intermittent and distending, moderate in severity, worsening in the supine position and slightly relieved in the sitting position. It was accompanied by a sensation of rectal tenesmus, fatigue, sweating, and five episodes of diarrhea (yellow, loose stools). There was no nausea, vomiting, hematemesis, melena, fever, dizziness, headache, or vaginal bleeding or discharge. She subsequently sought treatment at a local clinic, where she received one day of intravenous antibiotics (specific medication and dosage unknown). The abdominal pain failed to resolve. Four hours prior to admission, the patient felt the abdominal pain had intensified, becoming severe, though still tolerable. It was accompanied by abdominal distension and rectal tenesmus. She was subsequently admitted urgently on February 17, 2025. Past history: No history of uterine surgery. No history of Cesarean section.

Key physical examination findings on admission (February 17, 2025): T: 36.3°C, P: 140 bpm, R: 19 breaths/min, BP: 99/70 mmHg. She presented with an acute ill appearance, painful expression, could not maintain a comfortable position voluntarily, was conscious, and had cold, clammy extremities. Heart rate was 140 bpm. Chest examination revealed no significant abnormalities. The abdomen was flat, with marked tenderness, rebound tenderness, and muscle guarding throughout. Gynecological examination: A small amount of brownish, purulent discharge was present in the vagina, without foul odor. The cervix was normal sized, with marked cervical motion tenderness. The posterior fornix was full. The uterus was anteverted, irregularly enlarged to the size of a 2–3 month pregnancy, with marked uterine tenderness. Bilateral adnexal tenderness was significant. Culdocentesis yielded 5 ml of purulent fluid.

### 2.3 Key auxiliary examinations

Laboratory tests: Complete Blood Count (2025-2-17): White Blood Cells: 11.19 × 10^9^/L, Segmented Neutrophil Percentage: 86.6%, Hemoglobin: 119 g/L. CRP: 218.23 mg/L. Procalcitonin (PCT): 13.30 ng/ml. Arterial Blood Gas Lactate: 2.4 mmol/L. Laboratory indicators such as BNP, cardiac enzyme profile, routine coagulation tests, liver function, and renal function were within normal ranges. Ascitic fluid culture and identification: Moderate growth of Escherichia coli. Drug sensitivity testing showed susceptibility to Imipenem-Cilastatin Sodium, Piperacillin-Tazobactam Sodium, and Ceftazidime.

**Figure 1:**
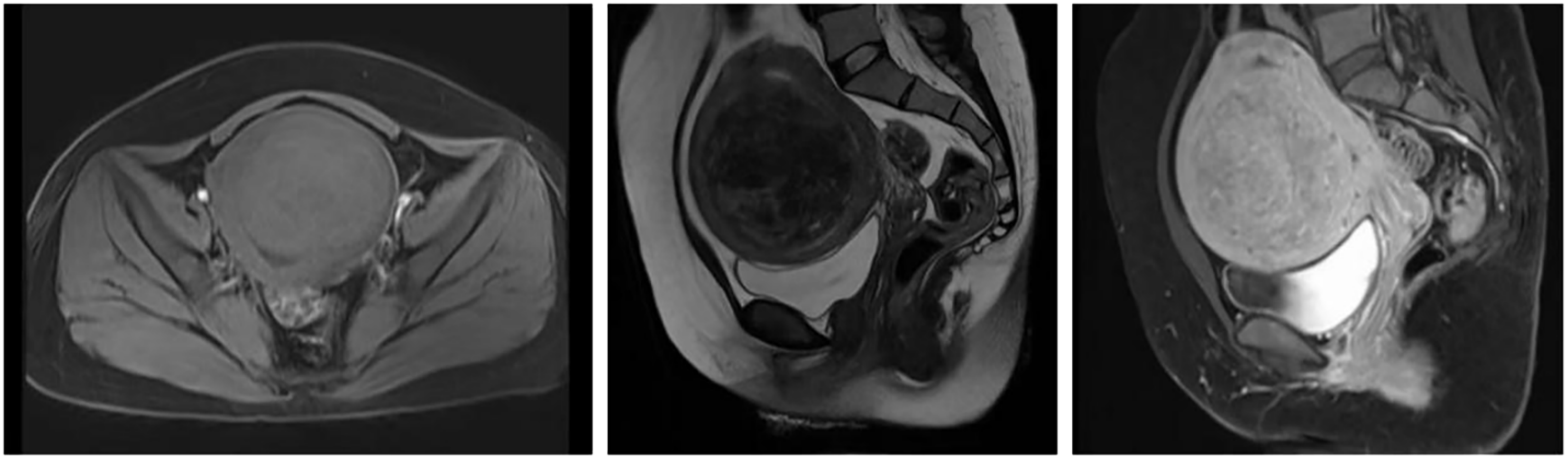
MRI Scan of Uterine Fibroids. Pelvic MRI scan obtained on December 24, 2024, prior to HIFU treatment, showing an enlarged uterus with a submucosal fibroid (8.7 × 8.1 × 9.0 cm) in the anterior uterine wall. The lesion appears iso- to hypointense on T1-weighted imaging and shows mixed slightly high and low signal intensity on T2-weighted and fat-suppressed sequences. Post-contrast images reveal heterogeneous enhancement slightly lower than that of the myometrium.

Imaging examination: Pelvic enhanced MRI findings dated December 24, 2024 (prior to High-Intensity Focused Ultrasound treatment): The uterine volume was enlarged, with a submucosal fibroid (8.7 × 8.1 × 9.0 cm) located in the anterior wall, compressing the endometrium and bladder (**Figure 1**).

Combined transabdominal and transvaginal color Doppler ultrasound examination on February 17, 2025: The uterus is anteverted, with an anteroposterior diameter of the uterine body measuring approximately 7.5 cm. The parenchymal echogenicity is heterogeneous, and the endometrium is not clearly visualized. A heterogeneous hypoechoic area measuring approximately 6.6 × 5.4 × 4.9 cm is seen within the uterine wall, exhibiting poorly-defined borders and no significant blood flow signal. Fluid-filled dark areas were observed in the pelvic cavity, with a maximum anteroposterior depth of about 2.7 cm, showing poor acoustic transmission. Fluid-filled dark areas were seen in the lower abdominal cavity, with a maximum anteroposterior depth of about 2.8 cm, showing poor acoustic transmission.

**Figure 2:**
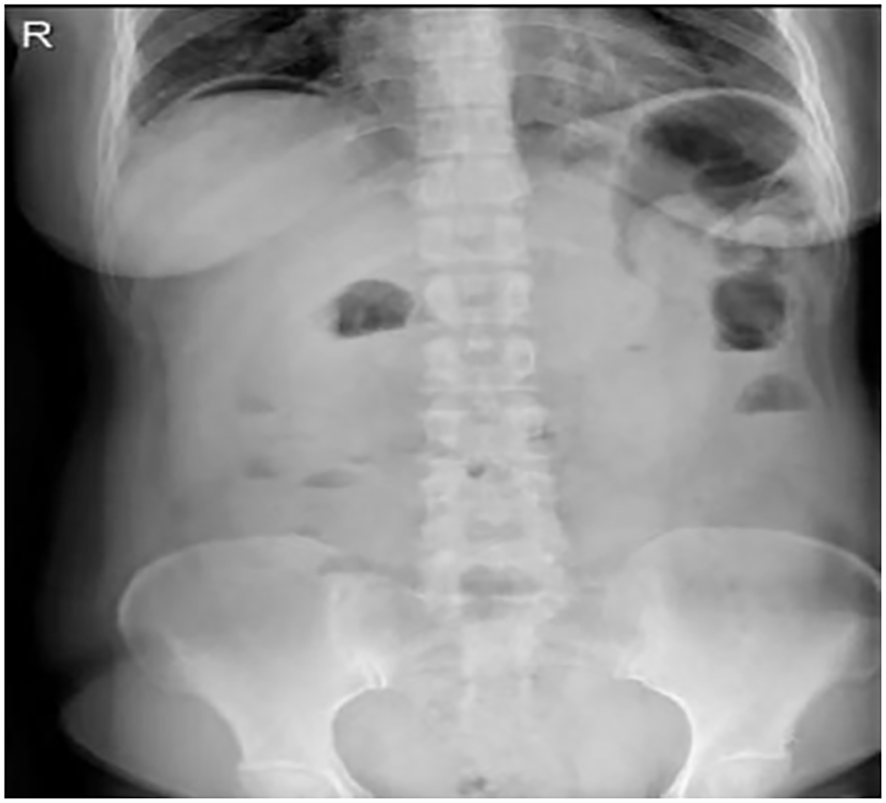
Abdominal erect view radiograph.

Abdominal erect view X-ray examination on February 17, 2025: 1. An arc-shaped gas density shadow is observed under the right hemidiaphragm, suggestive of hollow organ perforation. 2. Short air-fluid levels are seen in the mid-lower abdomen, indicating possible intestinal obstruction. See **Figure 2**.

**Figure 3:**
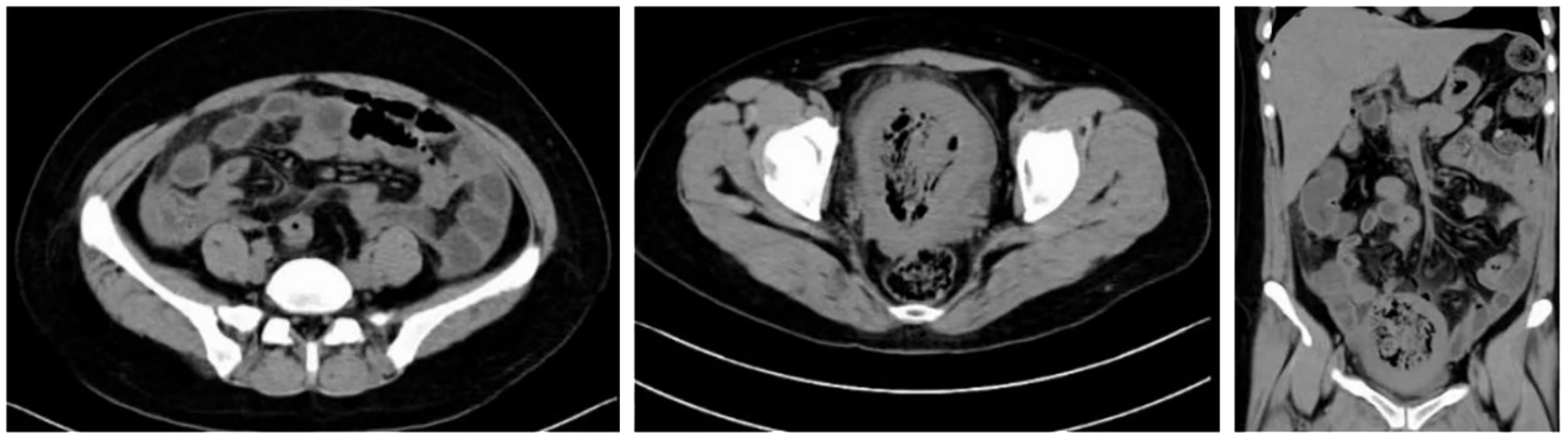
Abdominal and pelvic CT scan images.

Non-contrast abdominal and pelvic CT scan performed on February 17, 2025: The uterus is significantly enlarged with heterogeneous increased density within its cavity, demonstrating substantial gas accumulation. The uterine wall appears blurred, with localized irregularity and marked thinning of the fundal wall, accompanied by surrounding gas. Multiple free gas shadows are observed in the abdominal cavity, suggesting uterine perforation. A small amount of fluid is present in the abdominal and pelvic cavities. Haziness of the fat planes is noted in the abdominopelvic region, indicating inflammation. See **Figure 3**.

Admission diagnosis: 1. Uterine Rupture and Perforation. 2. Septic Shock.

## 3 Treatment method

**Figure 4:**
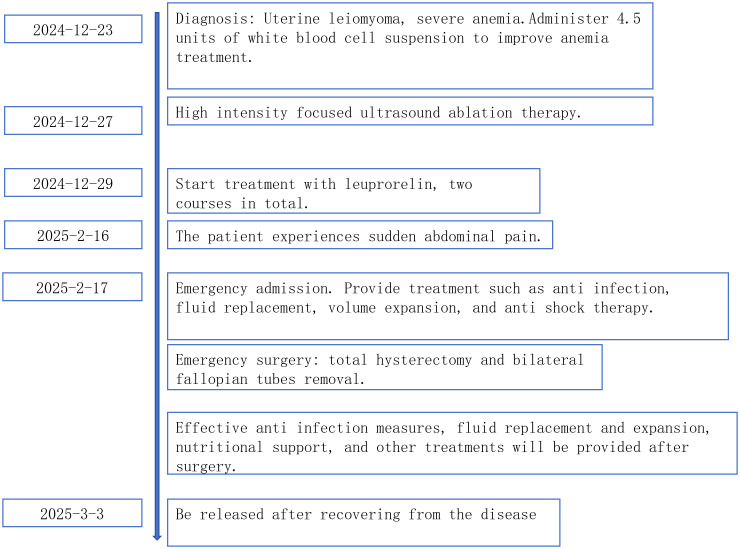
Timeline diagram of patient diagnosis and treatment.

On February 17, 2025, following admission, the patient was immediately administered Imipenem-Cilastatin Sodium for Injection (1g, intravenous infusion, q8h) for anti-infection treatment, along with intravenous fluid resuscitation for shock management and other symptomatic supportive therapies. An emergency diagnostic laparoscopy was performed. Intraoperative laparoscopic findings: Significant congestion was observed in the pelvic and abdominal cavities. Purulent exudate was widely adherent to the peritoneal surface, uterine surface, bladder surface, intestines, and epiploic appendages. A large amount of yellow purulent fluid was found in the pelvic cavity, paracolic gutters, interloop spaces, perihepatic area, and perisplenic area. Extensive filmy adhesions had formed between the intestinal loops, greater omentum, and the anterior abdominal wall/pelvic sidewalls. Filmy adhesions were present between the sigmoid colon, epiploic appendages, small intestine, bilateral adnexa, appendix, and the posterior uterine wall/necrotic fibroid lesion. The small intestines were matted together in a “cake-like” formation with mutual filmy adhesions. After lysis of adhesions, the uterus was found to be irregularly enlarged, approximately equivalent to the size of a 3-month gestation. In the anterior portion of the uterine body, a necrotic fibroid measuring about 8×8×7 cm was observed, exhibiting a grayish-white, rotten meat-like appearance. The tissue was friable with ill-defined boundaries between structures. The surface of the necrotic fibroid was covered with purulent exudate. The necrotic fibroid had perforated through the uterine serosal layer and was exposed within the abdominal cavity. Both fallopian tubes and ovaries were congested but showed no significant tortuosity or thickening. Due to the difficulty of the laparoscopic procedure, and after obtaining informed consent from the patient’s family, the surgery was converted to laparotomy. A total hysterectomy and bilateral salpingectomy were performed during the operation. Postoperative specimen examination: At the uterine fundus, the necrotic uterine fibroid was observed to have perforated through the serosal layer. A portion of the necrotic fibroid was protruding into the uterine cavity and had penetrated the endometrium. A small amount of yellow purulent fluid was present within the uterine cavity. The surgery was challenging, with an estimated intraoperative blood loss of about 100 ml. Postoperatively, the patient received the following anti-infective regimen: Imipenem-Cilastatin Sodium for Injection (1g, q6h, for 5 consecutive days); Piperacillin-Tazobactam Sodium for Injection (4.5g, q6h, for 6 consecutive days); Ceftazidime for Injection (1g, q8h, for 3 consecutive days). This was accompanied by appropriate fluid replacement and nutritional support therapy. After 14 days of treatment, the patient’s symptoms significantly improved and biochemical parameters returned to normal. The patient was discharged after recovery on March 3, 2025. The main laboratory indicators and their changes during the patient’s treatment course are shown in **Table 1**. The timeline of the patient’s primary diagnosis and treatment is shown in **Figure 4**.

**Table 1:** Main laboratory indicators and their changes during treatment.

Time	White blood cells (10^9^/L)	Segmented neutrophil percentage (%)	Hemoglobin (g/L)	High-sensitivity C-reactive protein (mg/L)	Procalcitonin (ng/ml)
2025/2/17	11.19	86.6	119	218.23	13.3
2025/2/18	10.92	92.4	83	243.25	4.378
2025/2/20	9.99	73.4	88	88.09	1.463
2025/2/22	17.71	77.2	106	94.69	0.541
2025/2/27	11.12	74.7	99	26.53	0.091
2025/3/2	6.27	68.6	105	9.31	0.025

## 4 Discussion

HIFU generates high temperatures (60–100 °C) by focusing ultrasonic waves, inducing coagulative necrosis in the target tissue while preserving surrounding normal structures (12, 13). Most current studies consider HIFU complications to be minor, with severe complications like uterine rupture being rare (14). This case report describes a rare complication in a 42-year-old female who developed uterine rupture, generalized peritonitis, and septic shock after undergoing HIFU ablation for uterine fibroids, ultimately requiring a total hysterectomy. This case not only highlights the potential risks of HIFU therapy but also serves as an important warning for clinical practice regarding patient selection, technical operation, and postoperative management.

Analyzing this case, the possible reasons leading to uterine rupture may include the following: 1) Large fibroid volume (8.7×8.1×9.0 cm in this case): This may lead to uneven thermal field distribution, particularly incomplete ablation at the periphery of the lesion. Residual viable tissue might form weak areas due to local ischemia or inflammatory response. In this case, the post-operative finding that the necrotic fibroid tissue penetrated the uterine serosa and was exposed in the abdominal cavity suggests the ablation depth or extent might have exceeded expectations, or the reparative capacity of the myometrium was compromised. 2) Subserosal location of the fibroid: Post-HIFU tissue edema and necrotic liquefaction could increase tension within the uterine wall. Changes in intra-abdominal pressure might then potentially induce perforation (9). 3) Post-HIFU Leuprolide therapy: This drug shrinks fibroids by suppressing estrogen secretion, but it might delay uterine tissue repair (15), potentially increasing the risk of uterine rupture. 4) Infection as a contributing factor: Infection can cause congestion and edema in the lesion and surrounding tissues, significantly increasing the tension within the necrotic fibroid tissue, potentially leading to tearing of the uterine serosa. Previous literature also reports cases of uterine rupture during pregnancy after HIFU treatment (14).

The development of septic shock in this patient involved multiple factors. Uterine rupture allowed direct exposure of necrotic tissue to the peritoneal cavity, providing an ideal environment for bacterial colonization. The reduced local blood supply to the uterus post-HIFU might weaken immune defenses. Additionally, the patient’s self-administration of amoxicillin and empirical antibiotic treatment at a local clinic might have masked early signs of infection, potentially contributing to increased pathogen drug resistance. Furthermore, the patient’s pre-existing anemia likely increased susceptibility to infection.

After admission, diagnosis was rapidly confirmed via laparoscopic exploration. However, due to severe pelvic adhesions, the procedure was converted to laparotomy, culminating in a total hysterectomy. This decision aligned with the principle of source control for infection but also reflects that HIFU treatment might increase the risk of pelvic adhesions (16), potentially complicating laparoscopic surgery. The postoperative antibiotic regimen employing imipenem and piperacillin-tazobactam provided coverage for Gram-negative bacteria and anaerobes, consistent with guidelines for empirical treatment of intra-abdominal infections. Based on this case, the authors suggest a need to explore strategies for optimizing specific protocols for preventing infection after HIFU, such as pre-operative screening for occult infections, strict aseptic technique during the procedure, and considering short-term post-operative antibiotic prophylaxis. In recent years, several studies have re-evaluated the safety profile and long-term outcomes of HIFU. Recent systematic reviews (4, 11, 14) have reported that although the overall incidence of severe complications remains low, cases of uterine rupture, infection, and reintervention highlight the importance of standardized procedural protocols and patient selection criteria.

## 5 Conclusion

Although HIFU is widely regarded as a safe and minimally invasive technique for treating uterine fibroids, severe complications such as uterine rupture and septic shock can still occur. This case suggests that the following factors may increase the risk of severe complications: excessively large fibroid volume, subserosal location of the fibroid, cumulative treatment energy exceeding the tissue tolerance threshold, and infection of the treated lesion post-HIFU. Therefore, strict adherence to HIFU indications is crucial. As a case report, the conclusions of this study are limited by its single-subject nature and cannot establish causality. The specific incidence of uterine rupture following HIFU requires support from large-sample, long-term follow-up data.

## Data Availability

The raw data supporting the conclusions of this article will be made available by the authors, without undue reservation.
